# Subgroup J avian leukosis virus infection inhibits autophagy in DF-1 cells

**DOI:** 10.1186/1743-422X-10-196

**Published:** 2013-06-17

**Authors:** Haixia Liu, Weisheng Cao, Yuhao Li, Min Feng, Xiaochan Wu, Kangzhen Yu, Ming Liao

**Affiliations:** 1 College of Veterinary Medicine, South China Agricultural University, Guangzhou, People’s Republic of China; 2 MOA Key Laboratory of Animal Vaccine Development, Guangzhou, People’s Republic of China; 3 Ministry of Agriculture of the People’s Republic of China, Beijing, People’s Republic of China

**Keywords:** Autophagy, ALV-J, LC3, atg5

## Abstract

**Background:**

Subgroup J avian leukosis virus (ALV-J) infection can induce tumor-related diseases in chickens. Previous studies by our laboratory demonstrated that ALV-J infection of DF-1 cells resulted in altered activity and phosphorylation of AKT. However, little is known about the subsequent activation of host DF-1 cells.

**Results:**

In the current study, autophagy inhibition was observed for ALV-J infected DF-1 cells. Our data showed that the autophagosome protein, microtubule-associated protein 1 light chain 3-II (LC3-II), was reduced considerably in DF-1 cells infected with active ALV-J, while no change was observed for cells infected with inactivated ALV-J. Autophagy inhibition was also confirmed by fluorescence microscopy and transmission electron microscopy. Interestingly, when autophagy was promoted by rapamycin, the titers of ALV-J replication were decreased, and the replication level of ALV-J was significantly enhanced when atg5 (autophagy-related gene 5) was knocked out.

**Conclusions:**

These results suggested that ALV-J infection could down-regulate autophagy in DF-1 cells during viral replication. This study is the first to report on the relationship between ALV-J infection and autophagy in DF-1 cells.

## Background

Avian leukosis viruses (ALV) are avian retroviruses responsible for inducing tumor-related diseases in poultry. ALV has the potential to cause significant economic losses to the poultry industry in many countries, including China. Chicken ALVs are classified into six subgroups according to antigenic differences in the surface envelope glycoprotein; endogenous viruses belong to subgroup ALV-E while all exogenous viruses belong to subgroups A–D and J [1]. Since its first isolation from meat-type chickens in the early 1990s, ALV-J has induced a high incidence of myeloid tumors in some chicken lines and their hybridizing passages [2]. Sequence analysis revealed that ALV-J arose from recombination between one or more endogenous and exogenous ALVs [3,4]. ALV-J tumor-related diseases are not restricted to commercial meat-type poultry, with reports of egg-type poultry and some local Chinese chicken breeds also being affected. ALV-J can induce various grossly visible tumors in chickens, especially the widespread pathotypes of myeloid leukosis and hemangioma, responsible for regional epidemics [2].

ALV shares a similar replication cycle to other retroviruses such as human immunodeficiency virus type-1 (HIV-1). In brief, ALV-J gains entry to the host cell through the interaction of its envelope glycoprotein with the Na^+^/H^+^ exchanger type 1 (cNHE1) receptor of the cell [5]. Once inside, the cell virus replication proceeds in the following manner; reverse transcription, integration of viral DNA into the host genome, RNA synthesis, translation, particle assembly, virus maturation, and release of virions from the cell by budding. Retroviruses have developed a number of strategies using cellular signaling pathways to assist in their replication in host cells [6]. Our previous study demonstrated that ALV-J infection of DF-1 cells correlated with the activity and phosphorylation of AKT [7]. The activation of the PI3K/AKT signaling pathway by infection with ALV-J is known to play an important role in virus entry, but the relationship between ALV-J replication and functional changes in host cells is yet to be elucidated.

Autophagy is an intracellular biological process in which damaged organelles and long-lived proteins are encapsidated and directed to lysosomes for degradation [8]. The degradation products can then be reused by cells, bacteria or viruses. The mechanism of autophagy was originally studied in yeast [9] and to date more than 31 autophagy-related genes have been identified. Several homologues have also been identified in mammals. Increasing numbers of studies have shown that autophagy is involved in cancer, muscular discord and neurodegeneration, major histocompatibility complex (MHC) antigen presentation, and innate immunity against certain bacteria and viruses [10-13]. Autophagy can be activated by some bacterial or viral infections [14-17], while it is inhibited by others such as HIV-1 and Human cytomegalovirus [18,19]. Autophagy is regulated by several complex signaling pathways. For example, the class III PI3K/Beclin signaling pathway enhances autophagosome formation, while the class I PI3K/Akt/mTOR signaling pathway inhibits its formation [20,21].

Our previous study demonstrated that exogenous avian leukosis viruses activated the PI3K/AKT signal pathway in infected cells. AKT is known to be an upstream signaling molecule of autophagy, mediating its inhibitory effect. In the current study, we investigated whether ALV-J inhibited autophagy, and if so, the potential signaling pathway.

## Results

### Autophagy is down-regulated in ALV-J infected DF-1 cells

**Figure 1:**
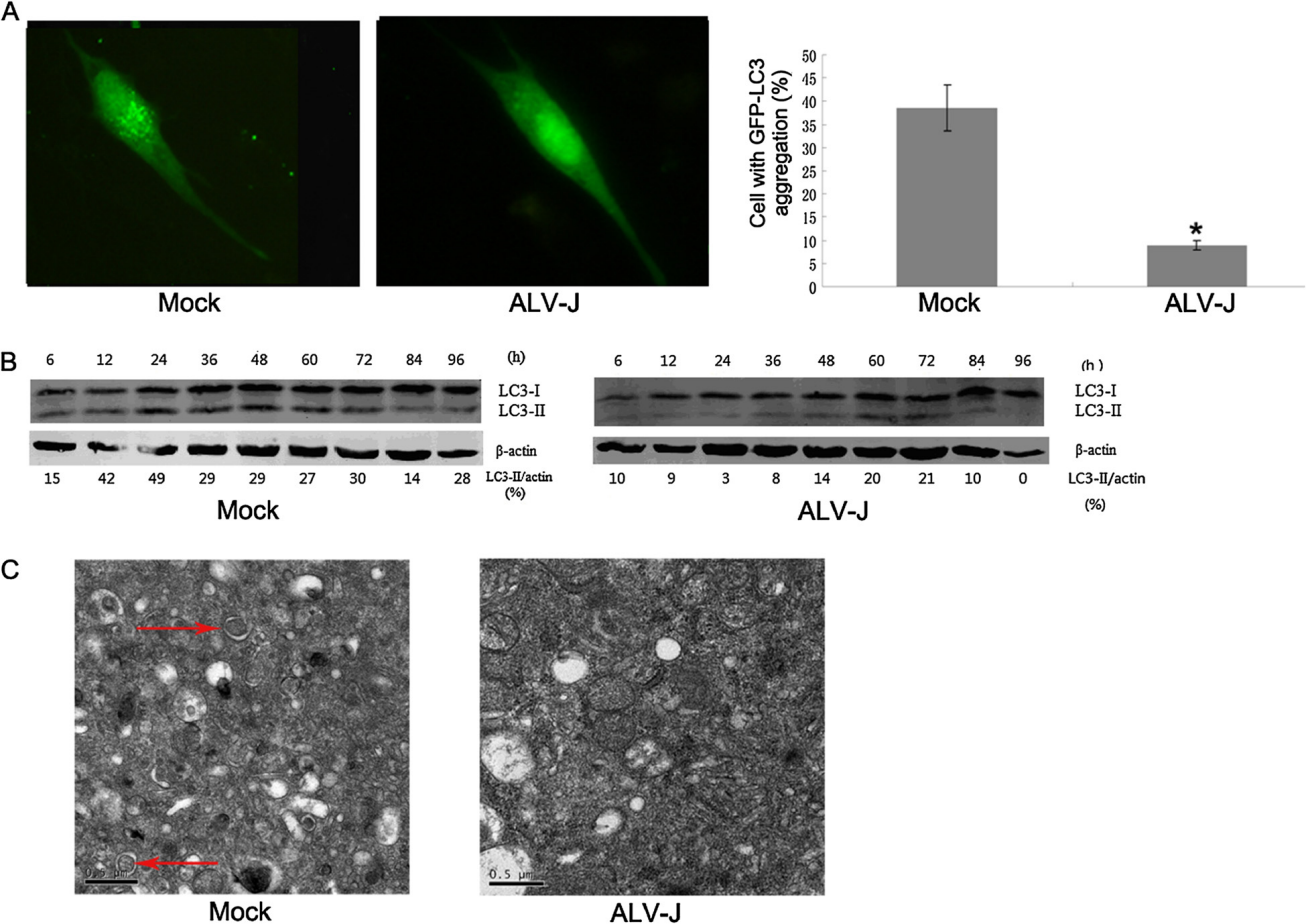
**ALV-J infection inhibits autophagy in DF-1 cells.** (**A**) Fluorescence microscopy revealed limited numbers of GFP-LC3 puncta in ALV-J-infected cells. DF-1 cells were transfected with GFP-LC3, infected with mock or ALV-J strain HN06 at an MOI of 1 for 2 h, and cultured in DMEM (+10% FBS) for 48 h. Cells with punctuated GFP-LC3 were counted and displayed as a histogram. * *P*-value < 0.05. (**B**) DF-1 cells were infected with mock or ALV-J (MOI = 1), harvested at 6, 12, 24, 36, 48, 60, 72, 84 and 96 h p.i., the cells lysed and proteins analyzed by western blotting. (**C**) Autophagosomes were observed with transmission electron microscopy. Mock-infected cells showed autophagosomes with double-membranes and autophagic vacuoles. No autophagic vacuoles were observed for ALV-J infected cells (MOI = 1).

ALV-J inhibition of autophagy was determined by examining autophagosome formation in DF-1 cells. Autophagy induction is often characterized by the appearance of increasing cytoplasmic puncta. As shown in Figure 1A, GFP-LC3 puncta were significantly reduced in ALV-J-infected cells when compared with mock-infected cells. Based on these results, we postulated that autophagy induction was inhibited in ALV-J infected DF-1 cells.

Further confirmation of autophagy inhibition by ALV-J infection was established from autophagy levels, as determined by the ratio of LC3 II/β-actin (Figure 1B). Western blot analysis revealed that from 6 to 96 h post-infection (h p.i.), ALV-J-infected cells had remarkably lower LC3 II/β-actin ratios compared with the corresponding mock-infected cells. The lowest LC3II/β-actin ratio was observed at 24 h p.i. for ALV-J infected cells (Figure 1B).

The images obtained by transmission electron microscopy are presented in Figure 1C. These results demonstrated that phagophores (double-membraned autophagosomes) were present in mock-infected cells, while comparatively few were observed for ALV-J-infected cells.

**Figure 2:**
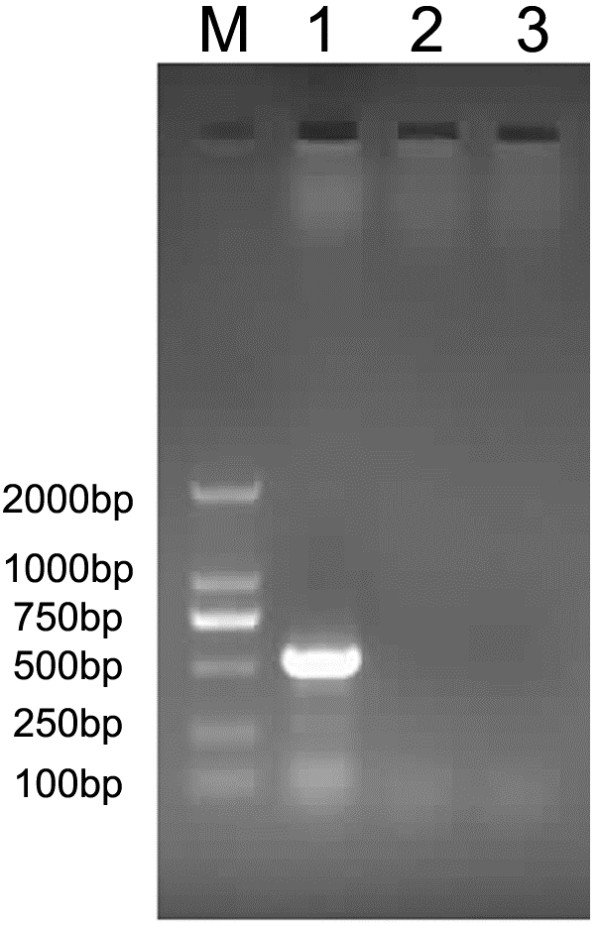
**PCR confirmation of ALV-J inactivation.** The ALV-J specific band was approximately 500 bp in length. M: DL2000 Marker, 1. ALV-J, 2 Inactivated ALV-J, 3. Mock.

**Figure 3:**
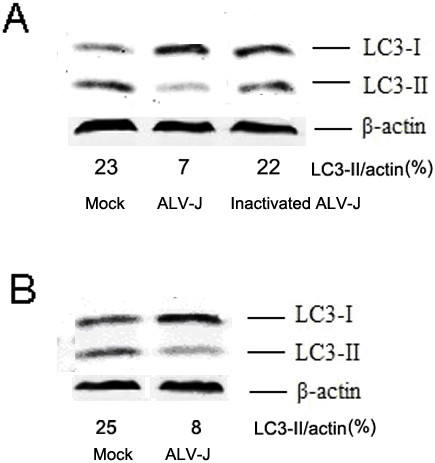
**Autophagy is inhibited by ALV-J replication.** (**A**) DF-1 cells were infected with mock, ALV-J (MOI = 1) or inactivated ALV-J. At 48 h p.i., the cells were analyzed by western blotting. (**B**) Autophagy was inhibited in ALV-J replicating cells by rapamycin. Following 24 h infection with mock or ALV-J (MOI = 1), cells were treated with rapamycin (200 nM) for 2 h, prior to analysis by western blotting.

To investigate whether autophagy inhibition was caused by ALV-J replication, DF-1 cells were infected with mock, ALV-J or heat-inactivated ALV-J. PCR analysis confirmed the heat inactivation of ALV-J (Figure 2). Western blot analysis confirmed greater levels of LC3 II protein for both mock and inactivated ALV-J-infected cells than for ALV-J-infected cells (Figure 3A). These results suggested that ALV-J infection and replication may have multiple effects on autophagy.

Collectively, these results revealed that ALV-J replication inhibited autophagy, even in the presence of rapamycin. As shown in Figure 3B, ALV-J-infected cells induced by rapamycin (200 nM) were unable to attain the autophagy levels observed for mock-infected cells.

### Pharmacological induction of autophagy decreases the yield of ALV-J

**Figure 4:**
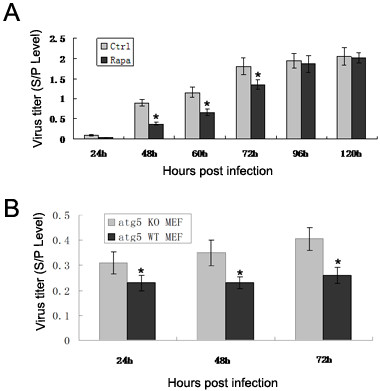
**Regulation of ALV-J-inhibited autophagy in DF-1 cells affects virus production.** (**A**) DF-1 cells were pretreated with or without rapamycin (200 nM) for 2 h, then infected with ALV-J (MOI = 0.1). The virus yield was determined at the indicated times. (**B**) atg5 KO/WT MEF cell lines were infected with ALV-J (MOI = 0.1). The virus yield was determined at the indicated times. * *P*-value < 0.05.

The effect of autophagy on ALV-J replication was evaluated by measuring ALV-J progeny virus titers following autophagosome induction by rapamycin. As shown in Figure 4A, extracellular virus titers were significantly reduced for DF-1 cells treated with rapamycin (200 nM). Viral titers of mock-treated cells were higher than for rapamycin-treated cells from 1–5 d p.i. From 48–72 h p.i., autophagy induction significantly decreased the yield of ALV-J progeny.

### Depletion of atg5 increases ALV-J replication

During autophagy, autophagosome formation requires the participation of several activated autophagy-related gene proteins (Atg), such as Atg5 [22]. To establish the role of atg5 in ALV-J replication, atg5 KO/WT MEF cell lines were used. The results demonstrated that the level of ALV-J replication in atg5 KO MEF cells was significantly higher than for atg5 WT MEF cells (Figure 4B).

**Figure 5:**
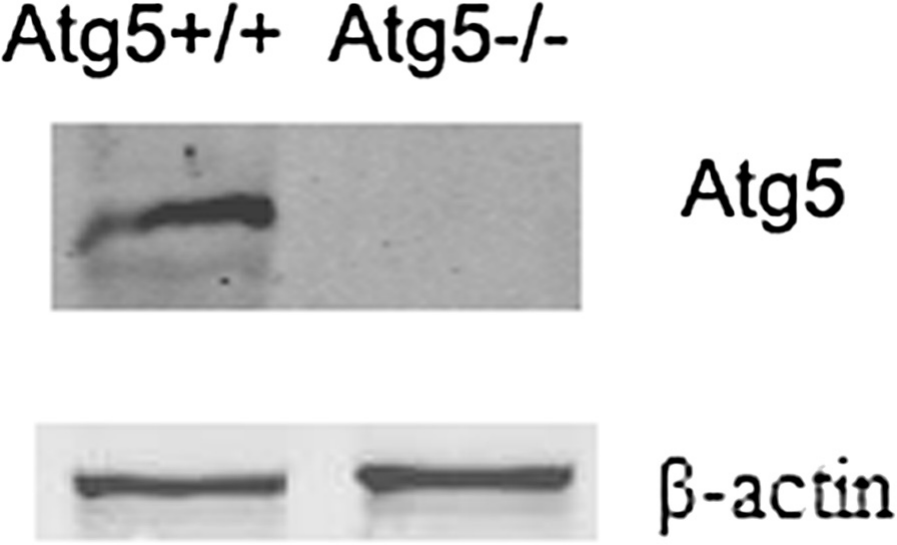
**Verification of atg5**^**+/+ **^**MEFs and atg5**^**-/- **^**MEFs.** Following 48 h culture, atg5^+/+^ MEFs and atg5^-/-^ MEFs were collected for western blot analysis using Atg5 rabbit monoclonal antibody. The Atg5 protein has an approximate M.W. of 55 kDa. The absence of this 55 kDa band confirmed Atg5 knockdown in atg5^-/-^ MEFs. In contrast, this band was observed for atg5^+/+^ MEFs.

Western blot analysis verified the presence of the 55 kDa Atg5 protein in WT MEF cells and similarly, its absence for atg5 KO MEF cells (Figure 5).

## Discussion

To date, we are the first group to report that ALV-J infection down-regulates autophagy in DF-1 cells during acute infection. This result is in accordance with autophagy studies conducted for another *Retrovirdiae* virus, HIV [23]. In the current study, autophagy protein LC3 II levels were found to be consistently lower during ALV-J infection and replication than for mock-infection. The lowest LC3II/actin ratio was observed at 24 h p.i.; consistent with the time required for one replication cycle of ALV-J [23]. Moreover, the level of ALV-J replication in MEF and atg5^-^/^-^ MEF cells further confirmed this result.

In recent years, human and animal Atgs have been identified, and interrelated pathways involved in autophagy have been uncovered [24]. Autophagy not only contributes to the maintenance of cellular homeostasis, it also acts as an innate host defense mechanism against microbial invasion [20,25,26]. To counteract host autophagy defense mechanisms, some bacteria and viruses have evolved strategies to antagonize this process. For example, autophagy is activated during infection with influenza A virus, herpes simplex virus type 1 (HSV-1), hepatitis C virus (HCV) and some bacteria [16,17,27]. These microorganisms may use proteins of the autophagic pathway to facilitate their replication. Conversely, some viruses such as human cytomegalovirus (HCMV) and immunodeficiency virus type-1 (HIV-1) have developed strategies to inhibit autophagy during infection [18,20].

Autophagy protein Atg5 promotes autophagy, and is critical for autophagosome formation. Atg5 is cleaved by calpain into a 24 kDa truncated peptide that mediates apoptosis, but with a concomitant loss in its autophagic property. Atg5 represents a molecular switch between autophagy and apoptosis [28,29]. We found that ALV-J replicated to a higher level in atg5 KO MEF than in atg5 WT MEF, while ALV-J infection of DF-1 cells demonstrated an important role for atg5 in ALV-J replication.

As both HIV-1 and ALV-J belong to the family *Retroviridae*, one could assume that they may share similarities in their replication life cycle in host cells. HIV-1 infection can inhibit autophagy [18] and activate mTOR, which is known to play an important role in gp120-induced apoptosis [30,31]. We previously showed that ALV-J infection activated the PI3K-AKT signaling pathway for virus entry and replication [7]. mTOR as a downstream effector in the PI3K-AKT-mTOR signaling pathway plays an important role in autophagy and apoptosis. Rapamycin-induced blocking of mTOR activity inhibited ALV-J replication, and rapamycin failed to induce autophagy in ALV-J-infected cells. These results suggested that the down-regulation of autophagy during ALV-J infection and replication is mediated through the activation of mTOR.

In conclusion, ALV-J infection down-regulates autophagy to facilitate viral replication, potentially through the PI3K/AKT/mTOR signaling pathway. Further studies on the precise molecular mechanisms of autophagy and ALV-J replication are needed to clarify the role of autophagy in ALV-J pathogenesis and tumorigenesis.

## Methods

### Cell culture and virus infection

Chicken DF-1 [32], MEF and atg5^-^/^-^ MEF cells [28] were grown in Dulbecco’s modified Eagle medium (DMEM) supplemented with 10% fetal bovine serum (FBS) at 37°C in a humidified atmosphere containing 5% CO_2_. DF-1 cells were inoculated with ALV-J strain HN06, and the cell culture supernatant collected at 7 days post-inoculation (d p.i.). Viral titers were established by ELISA according to the manufacturer’s instructions (ALV-J Ag Test, IDEXX, USA). The virus was identified using monoclonal anti-gp85 antibody of ALV-J provided by professor Ai-jian Qin, Yangzhou University, Jiangsu Province, China. Cell culture supernatant was aliquoted and stored as viral stocks at -80°C until use. Inactivated ALV-J was prepared by heating virus stocks for 1 h at 56°C, as described previously [7].

### Cell transfection and fluorescence microscopy

Once DF-1 cells had attained 80% confluency, they were transfected with GFP-LC3 using Lipofectamine 2000 (Invitrogen), according to the manufacturer’s instructions. At 12 h post-transfection, cells were either mock-infected or infected with ALV-J strain HN06 at an M.O.I of 1. At 48 h post-infection, cells were analyzed by fluorescence microscopy. The plasmid GFP-LC3 was kindly provided by Professor Chan Ding (Shanghai Veterinary Research Institute, Shanghai, China).

### Transmission electron microscopy

The observation of autophagosome formation by transmission electron microscopy is considered as the gold standard. At 48 h post mock or ALV-J strain HN06 infection, DF-1 cells were washed three times, trypsinized, and collected by centrifugation. The cell pellets were fixed with 2.5% glutaric dialdehyde for 30 min, post-fixed with OSO_4_ in cacodylate buffer for 1 h, and dehydrated stepwise with ethanol. The dehydrated pellets were rinsed with propylene oxide for 30 min, and embedded in Spurr resin for sectioning [29]. Thin sections were cut and observed under a transmission electron microscope (Tecnai 12, FEI, Holland).

### Inactivation of ALV-J and detection by PCR

The protocol for heat inactivation and PCR detection of ALV-J was as reported elsewhere (Feng et al., 2011). In brief, ALV-J was inactivated by heating at 56°C for 1.5 h. DF-1 cells were infected with inactivated ALV-J, the cells recovered at 24 h post-infection, genomic DNA extracted, and detection achieved by PCR using ALV-J specific primers.

### Western blot analysis

DF-1 cells were harvested from 60 mm dishes at nominated times (6, 12, 24, 36, 48, 60, 72, 84, 96 h) post-infection. The cells were washed twice with PBS at 4°C, lysed on ice for 15 min, and clarified by centrifugation at 10,000 × *g* for 5 min at 4°C. Western blotting was performed as described previously [33].

To investigate the relationship between inactivated ALV-J and autophagy, DF-1 cells were infected with mock, ALV-J or inactivated ALV-J. At 48 h p.i., the cells were harvested and the proteins investigated by western blotting.

To investigate whether the replication of ALV-J may proceed through mTOR molecular pathways, the following experiment was performed. After 24 h infection with either mock or ALV-J (M.O.I. = 1), the cells were treated with rapamycin (200 nM) for 2 h, prior to collection for western blotting analysis.

The polyclonal rabbit anti-LC3 antibody (Sigma-Aldrich, St. Louis, MO, USA), an Atg5 rabbit monoclonal antibody (Abcam, USA) and a polyclonal antibody against β-actin (Sigma-Aldrich) were used according to the manufacturer’s instructions. Densitometric analysis of protein expression was carried out using Odyssey Application Software Version 3.0 (LI-COR Biosciences, NE, USA). All western blots were performed in triplicate for each experimental condition.

### Quantification of virus titer

Once DF-1 cells had attained 80% confluency, they were treated with rapamycin (200 nM) for 2 h prior to inoculation with ALV-J strain HN06 at an MOI of 0.1. At nominated times post-infection (24, 48, 60, 72, 96 and 120 h), cell culture supernatants were collected, and the virus titers determined by ELISA (ALV-J Ag Test, IDEXX, USA) according to the manufacturer’s instructions. All experiments were performed in triplicate.

MEF and atg5^-/-^ MEF cells were grown to approximately 80% confluency prior to inoculation with ALV-J strain HN06 at MOI of 0.1. At nominated times post-infection (1, 2, 3 d) the cell culture supernatants were collected, and the virus titers established by ELISA (ALV-J Ag Test, IDEXX) following the manufacturer’s instructions. All experiments were performed in triplicate.

### Statistical analysis

Data were analyzed using SPSS (version 10.0; Inc., Chicago, IL, USA). Independent sample T-tests were used to determine the statistical significance between samples. A *P*-value < 0.05 was considered to be statistically significant.

## Competing interests

The authors declare that they have no competing interests.

## Authors’ contributions

HL, WC and ML contributed to the concept and design of the study. KY, YL and MF contributed to data acquisition and analysis. XW performed the western blot assay. HL and WC wrote the manuscript. All authors have read and approved the final manuscript.
